# ‘…a paper …I hold to be great guns’: a commentary on Maxwell (1865) ‘A dynamical theory of the electromagnetic field’

**DOI:** 10.1098/rsta.2014.0473

**Published:** 2015-04-13

**Authors:** Malcolm Longair

**Affiliations:** Cavendish Laboratory, JJ Thomson Avenue, Cambridge CB3 0HE, UK

**Keywords:** Maxwell, electromagnetism, equations of the electromagnetic field, discovery of Maxwell's equations

## Abstract

Maxwell's great paper of 1865 established his dynamical theory of the electromagnetic field. The origins of the paper lay in his earlier papers of 1856, in which he began the mathematical elaboration of Faraday's researches into electromagnetism, and of 1861–1862, in which the displacement current was introduced. These earlier works were based upon mechanical analogies. In the paper of 1865, the focus shifts to the role of the fields themselves as a description of electromagnetic phenomena. The somewhat artificial mechanical models by which he had arrived at his field equations a few years earlier were stripped away. Maxwell's introduction of the concept of fields to explain physical phenomena provided the essential link between the mechanical world of Newtonian physics and the theory of fields, as elaborated by Einstein and others, which lies at the heart of twentieth and twenty-first century physics. This commentary was written to celebrate the 350th anniversary of the journal *Philosophical Transactions of the Royal Society*.

## 1. Prelude

On 16 June 1865, James Clerk Maxwell's paper ‘A dynamical theory of the electromagnetic field’ was sent to the printers Taylor and Francis for publication in *Philosophical Transactions of the Royal Society* [1].^1^ Six days earlier, on 10 June of the same year, Richard Wagner's *Tristan und Isolde* had its world première in the Court Theatre, Munich. The word *revolutionary* is the only adjective which begins to do justice to the extraordinary impact of these two events of 1865, one in the physical sciences, the other in music, opera and drama. Wagner's staggering innovations in musical harmony, structure and dramaturgy revolutionized the approaches of composers and profoundly influenced the music of the late nineteenth and twentieth centuries—music would never be the same again. In the same way, Maxwell's monumental paper laid the foundations for the innovations of twentieth century physics by placing fields at the heart of the theory of electromagnetism and of all subsequent fields which describe how matter and radiation behave at a fundamental level.

Einstein [2, p. 71] summed up Maxwell's achievement in 1931 on the occasion of the centenary of Maxwell's birth:

We may say that, before Maxwell, Physical Reality, in so far as it was to represent the process of nature, was thought of as consisting in material particles, whose variations consist only in movements governed by partial differential equations. Since Maxwell's time, Physical Reality has been thought of as represented by continuous fields, governed by partial differential equations, and not capable of any mechanical interpretation. This change in the conception of Reality is the most profound and the most fruitful that physics has experienced since the time of Newton.

Maxwell's achievements provided the essential bridge between the physics of Newton and of Einstein. The profound nature of this change in perception is beautifully articulated by Freeman Dyson [3, p. 4]:

Maxwell's theory becomes simple and intelligible only when you give up thinking in terms of mechanical models. Instead of thinking of mechanical objects as primary and electromagnetic stresses as secondary consequences, you must think of the electromagnetic field as primary and mechanical forces as secondary. The idea that the primary constituents of the universe are fields did not come easily to the physicists of Maxwell's generation. Fields are an abstract concept, far removed from the familiar world of things and forces. The field equations of Maxwell are partial differential equations. They cannot be expressed in simple words like Newton's law of motion, force equals mass times acceleration. Maxwell's theory had to wait for the next generation of physicists, Hertz and Lorentz and Einstein, to reveal its power and clarify its concepts. The next generation grew up with Maxwell's equations and was at home in a universe built out of fields. The primacy of fields was as natural to Einstein as the primacy of mechanical structures had been to Maxwell.

In this essay, I set Maxwell's paper of 1865 in the context of the evolution of his thinking about the nature of physical phenomena.

## 2. Act 1. Preliminaries

By the end of the eighteenth century, many of the basic experimental features of electrostatics and magnetostatics had been established. In the 1770s and 1780s, Charles-Augustin Coulomb performed very sensitive experiments which established directly the inverse square laws of electrostatics and magnetostatics, which at the time appeared to be quite separate physical phenomena. This changed with the development of the science of *current electricity*. In 1791, the Italian anatomist Luigi Galvani showed that electrical effects could stimulate the contraction of frogs' legs. In 1791, he showed that, when two dissimilar metals were used to make the connection between nerve and muscle in frogs' legs, the same form of muscular contraction was observed. This was announced as the discovery of *animal electricity*. Alessandro Volta suspected that the electric current was associated with the presence of different metals in contact with a moist body. In 1800, he demonstrated this by constructing a *voltaic pile*, which consisted of interleaved layers of copper and zinc separated by layers of pasteboard soaked in a conducting liquid. The most important aspect of Volta's experiments was that he had discovered a controllable source of electric current. The voltaic cell had a short lifetime as the pasteboard dried out and so he invented his *crown of cups*, in which the electrodes were placed in an electrolytic fluid in a series of glass vessels, as in modern batteries.

A key experimental advance was made in 1820 when Hans Christian Ørsted demonstrated that there is always a magnetic field associated with an electric current—this marked the beginning of the science of *electromagnetism*. Immediately, the physicists Jean-Baptiste Biot and Félix Savart set out to discover the dependence of the strength of the magnetic field at distance ***r*** from a current element of length d***l*** in which a current *I* is flowing. In SI notation, their answer, the *Biot–Savart law*, can be written
2.1
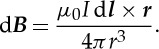

d***l*** is the length of the current element in the direction of the current *I* and ***r*** is measured from the current element d***l*** to the point at vector distance ***r***.

Next, André-Marie Ampère extended the Biot–Savart law to relate the current flowing though a closed loop to the integral of the component of the magnetic flux density around the loop. In modern vector notation, *Ampère's circuital law* in free space can be written
2.2
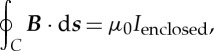

where *I*_enclosed_ is the total electric current flowing through the area enclosed by the loop *C*. In 1827, Ampère published his famous treatise [4], which included the demonstration that the magnetic field of a current loop could be represented by an equivalent magnetic shell. He also formulated the equation for the force between two current elements *d****l***_1_ and *d****l***_2_ carrying currents *I*_1_ and *I*_2_:
2.3
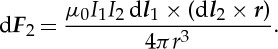

d***F***_2_ is the force acting on the current element d***l***_2_,***r*** being measured from d***l***_1_. Ampère demonstrated the relation between this law and the Biot–Savart law.

In 1827, Georg Simon Ohm formulated the relation between potential difference *V* and the current *I*, what is now known as *Ohm's law*, *V* =*RI*, where *R* is the resistance of the material through which the current flows. This was a controversial result until 1876 when Maxwell and his student George Chrystal established the law experimentally with high precision at the recently founded Cavendish Laboratory, of which Maxwell had been appointed the first Head and Cavendish Professor of Experimental Physics in 1871.

The above results were established by 1830 and comprise the whole of *static electricity*, the forces between *stationary* charges, magnets and currents. The essence of Maxwell's equations is that they also deal with *time-varying* phenomena. Over the succeeding 20 years, all the basic experimental features of time-varying electric and magnetic fields were established.

During the 1820s, many articles were submitted to the scientific journals describing electromagnetic effects and attempting to explain them. Michael Faraday was invited to survey this mass of experiment and speculation by the editor of the *Philosophical Magazine* and so began his systematic study of electromagnetic phenomena. These experiments led Faraday to introduce the key concept of *magnetic lines of force* to represent the direction in which the force acting upon a magnetic pole acts when placed in a magnetic field. The greater the number of lines of force per unit area in the plane perpendicular to the field lines, the greater the force acting upon the magnetic pole.

Believing firmly in the symmetry of nature, Faraday conjectured that, since an electric current could produce a magnetic field, it must also be possible to generate an electric current from a magnetic field. In 1831, he learned of Joseph Henry's experiments, in which very powerful electromagnets were used. Faraday immediately had the idea of observing the strain in the material of a strong electromagnet caused by the lines of force. He built a strong electromagnet by winding an insulating wire, through which a current could be passed, onto a thick iron ring, thus creating a magnetic field within the ring. The effects of the strain were to be detected by another winding on the ring, which was attached to a galvanometer to measure the amount of electric current produced.

The effect was not at all what Faraday might have expected. When the primary circuit was closed, there was a displacement of the galvanometer needle in the secondary winding—an electric current had been induced in the secondary wire through the medium of the iron ring. Deflections of the galvanometer were *only* observed when the current in the electromagnet was switched on and off. This was the discovery of *electromagnetic induction*. As early as 1831, Faraday had established the qualitative form of his law of induction in terms of the concept of lines of force—*the electromotive force induced in a current loop is directly related to the rate at which magnetic field lines are cut*, adding that

By magnetic curves, I mean lines of magnetic force which would be depicted by iron filings.

In 1834, Lenz enunciated the law which cleared up the issue of the direction of the induced electromotive force in the circuit—*the electromotive force acts in such a direction as to oppose the change in magnetic flux*.

Faraday could not formulate his theoretical ideas mathematically, but he was convinced that the concept of lines of force provided the key to understanding electromagnetic phenomena. In 1846, he speculated in a discourse to the Royal Institution that light might be some form of disturbance propagating along the field lines but the idea was received with considerable scepticism.

Faraday had an instinctive belief in the unity of the forces of nature, and in particular between light, electricity and magnetism. In 1846, he passed light through lead borate glass in the presence of a strong magnetic field. He demonstrated the phenomenon now known as *Faraday rotation*, in which the plane of polarization of linearly polarized light is rotated when the light rays travel along the magnetic field direction in the presence of a transparent dielectric. William Thomson, later Lord Kelvin, interpreted this phenomenon as evidence that magnetism was essentially rotational in nature and this was to influence strongly Maxwell's thinking about the physical nature of magnetic fields.

## 3. Act 2. Maxwell on ‘On Faraday's lines of force’ (1856)

Maxwell had displayed his talents as an experimental and theoretical physicist from an early age. His first degree in natural philosophy from Edinburgh University followed by his mathematical studies at Trinity College, Cambridge, as well as his omnivorous appetite for the most demanding texts of the great European mathematicians and natural philosophers, provided the ideal preparation for his researches into electromagnetism.^2^ As remarked by Sviedrys [13, p. 428],

[Maxwell] was one of the very few British physicists who combined the experimental and philosophical Scottish tradition with the mathematical training provided at Cambridge.

The distinctive feature of Maxwell's thinking was his ability to work by *analogy*. In 1856, he described his approach in an essay entitled *Analogies in Nature* written for the Apostles Club at Cambridge. Its essence can be caught from the following passage.

Whenever [men] see a relation between two things they know well, and think they see there must be a similar relation between things less known, they reason from one to the other. This supposes that, although pairs of things may differ widely from each other, the *relation* in the one pair may be the same as that in the other. Now, as in a scientific point of view the *relation* is the most important thing to know, a knowledge of the one thing leads us a long way towards knowledge of the other. [14, pp. 381–382]

In relation to electromagnetism, he found formal analogies between the mathematics of mechanical and hydrodynamical systems, and the phenomena of electrodynamics. He acknowledged throughout this work his debt to William Thomson, subsequently Lord Kelvin, who had made substantial steps in mathematizing electric and magnetic phenomena.

In the same year, 1856, Maxwell published the first of his series of papers on electromagnetism, ‘On Faraday's lines of force’ [15]. In the preface to his *Treatise on Electricity and Magnetism* of 1873 [16, p. viii], he recalled:

before I began the study of electricity I resolved to read no mathematics on the subject till I had first read through Faraday's *Experimental Researches in Electricity*.

The first part of the paper enlarged upon the technique of analogy and drew particular attention to its application to incompressible fluid flow and magnetic lines of force. For the sake of clarity, we will recast Maxwell's analysis in SI units using the vector operator expressions div, grad and curl. The use of vector methods was only introduced by Maxwell in his paper of 1870 entitled ‘On the mathematical classification of physical quantities’ [5,17]—he invented the terms ‘slope’ (now ‘gradient’), ‘curl’^3^ and ‘convergence’ (the opposite of ‘divergence’) to provide an intuitive feel for the meaning of these vector operators. In 1856, the partial derivatives were written out explicitly in Cartesian form. The mathematics of rotational fluid flow and the equivalents of div, grad and curl were familiar to mathematical physicists at the time. Thomson and Maxwell, for example, needed these tools to describe fluid flow in vortex ring models of atoms.

Maxwell started with the analogy between incompressible fluid flow and magnetic lines of force. The velocity ***u*** is analogous to the magnetic flux density ***B***. If the tubes of force, or steamlines, diverge, the strength of the field decreases, as does the fluid velocity. This enabled Maxwell to write down immediately the mathematical expression for the behaviour of magnetic fields in free space,
3.1
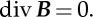

The same type of reasoning applies to electric fields in free space, but they can originate on charges and so there is a source term on the right-hand side
3.2
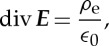

where ***E*** is the electric field strength and *ρ*_e_ is the electric charge density.

Faraday's law of electromagnetic induction was first put into mathematical form by Neumann in 1845. The induced electromotive force 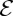 is proportional to the rate of change of magnetic flux through the circuit
3.3
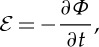

where *Φ* is the total magnetic flux threading the circuit. This is exactly equivalent mathematically to the more powerful vector equation which describes what happens at a point in space. Using Stokes' theorem,
3.4
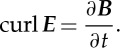



For currents and the magnetic fields they produce, there is a similar relation derived from Ampère's law:
3.5
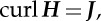

where ***H*** is the magnetic field strength and ***J*** is the current density.

The final achievement of this paper was the formal introduction of what is now called the *vector potential* ***A***. Such a vector had already been introduced by Neumann, Weber and Kirchhoff in order to calculate induced currents,
3.6
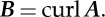

This definition is clearly consistent with (3.1), since div curl ***A***=0. Maxwell went further and showed how the induced electric field ***E*** could be related to ***A***. Incorporating the definition (3.6) into (3.4), we obtain
3.7
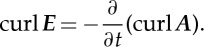

Interchanging the order of the time and spatial derivatives on the right-hand side, we obtain
3.8
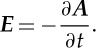



These analyses provide formal coherence for the phenomena of electromagnetism. Maxwell had obtained the primitive, but *incomplete*, set of equations for the electromagnetic fields, as represented by the relations (3.1), (3.2), (3.4) and (3.5). He still lacked, however, a physical model—for Maxwell, a mechanical analogue—for the behaviour of electromagnetic phenomena.

## 4. Act 3. Maxwell on ‘On physical lines of force’ (1861–1862)

Maxwell developed his solution in 1861–1862 in a series of papers entitled ‘On physical lines of force’ [18–21]. Since his earlier work on the analogy between ***u*** and ***B***, he had become more and more convinced that magnetism was essentially rotational in nature. His aim was to devise a model for the medium filling all space which could account for the stresses that Faraday had associated with magnetic lines of force—in other words, a mechanical model for the *aether*, which was assumed to be the medium through which light was propagated. In his intriguing book *Innovation in Maxwell's Electrodynamics* [22], David Siegel has carried out a detailed analysis of these papers, vividly demonstrating the richness of Maxwell's insights in drawing physical analogies between mechanical and electromagnetic phenomena.

The model was based upon the analogy between a rotating vortex tube and a tube of magnetic flux. The analogy comes about as follows. If left on their own, magnetic field lines expand apart, exactly as occurs in the case of a fluid vortex tube, if the rotational centrifugal forces are not balanced. In addition, the rotational kinetic energy of the vortices can be written
4.1
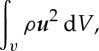

where *ρ* is the density of the fluid and ***u*** its rotational velocity. This expression is formally identical to that for the energy contained in a magnetic field distribution, 
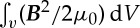
. Again ***u*** is analogous to ***B***—the greater the velocity of rotation of the tube, the stronger the magnetic field. Maxwell postulated that everywhere the local magnetic flux density is proportional to the angular velocity of the vortex tube, so that the angular momentum vector ***L*** is parallel to the axis of the vortex, and so parallel to the magnetic flux density vector ***B***.

Maxwell began with a model in which all space is filled with vortex tubes. There is, however, an immediate mechanical problem. Friction between neighbouring vortices would lead to their disruption. Maxwell adopted the practical engineering solution of inserting ‘idle wheels’, or ‘ball–bearings’, between the vortices so that they could all rotate in the same direction without friction. Maxwell's published picture of the vortices, represented by an array of rotating hexagons, is shown in figure 2. He then identified the idle wheels with electric particles which, if they were free to move, would carry an electric current as in a conductor. In insulators, *including free space*, they would not be free to move through the distribution of vortex tubes and so could not carry an electric current. I have no doubt that this rather extreme example of the technique of working by analogy was a contributory cause to ‘a feeling of discomfort, and often even of mistrust,…’ to which Poincaré alluded when French mathematical physicists first encountered the works of Maxwell [23].

**Figure 1.**
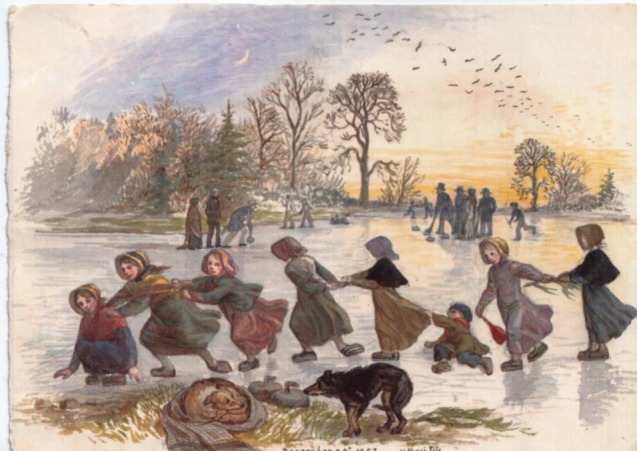
Watercolour by Jemima Wedderburn showing children playing on the frozen loch at St Mary's Isle near the family home at Glenlair on 23 December 1853. In the background, the adults are seen curling. (Courtesy of The Maxwell at Glenlair Trust and James Clerk Maxwell Foundation.)

**Figure 2.**
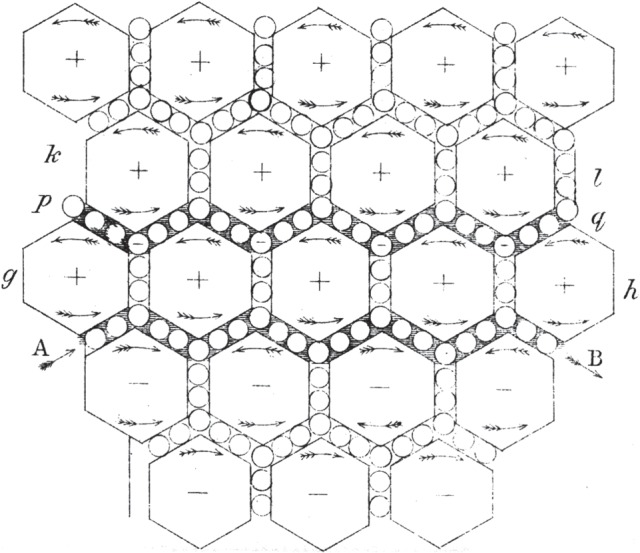
Maxwell's diagram from the *Philosophical Magazine* of 1861 showing the rotating vortices represented by hexagons and the idle wheels between them [18, plate V, fig. 2].

Remarkably, this mechanical model for the aether could account for all known phenomena of electromagnetism.^4^ As an example of induction, consider the effect of embedding a second wire in the magnetic field of a wire carrying a current *I*. If the current is steady, there is no change in the current in the second wire. If, however, the current changes, a rotational impulse is communicated through the intervening idle wheels and vortices and a reverse current is induced in the second wire.

Part III of the paper contains the flash of genius which led to the discovery of the complete set of Maxwell's equations. He now considered how insulators store electrical energy. He made the assumption that, in insulators, the idle wheels, or electric particles, can be *displaced* from their ‘fixed’ equilibrium positions by the action of an electric field. He then attributed the electrostatic energy in the medium to the *elastic potential energy* associated with the displacement of the electric particles. In his subsequent paper of 1865, he refers to this key innovation as *electric elasticity*.

This postulate had two immediate consequences. First, when the electric field applied to the medium is varying, there are small changes in the positions of the electric particles in the insulating medium or vacuum, and so there are small currents associated with this motion of the particles. In other words, there is a current ***J***_D_ associated with the *displacement* of the electric particles from their equilibrium positions. The equations had become:
4.2
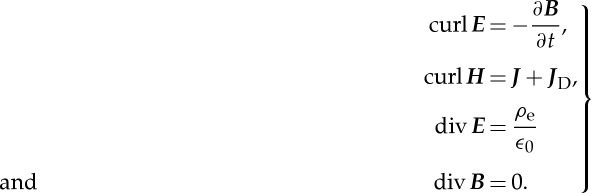



Second, by virtue of the electric particles being bound elastically, any disturbance results in the propagation of waves though the medium. Maxwell could then carry out a straightforward calculation to find the physical nature and speed *c* at which disturbances can be propagated through the insulator, or vacuum. To do this, he had to solve the modified version of the field equations of electromagnetism (4.2) in a vacuum. A simple version of the argument is given in appendix A. The answer was *c*=(*εε*_0_*μμ*_0_)^−1/2^, where *ε* and *μ* are the permittivity and permeability of the medium.

Notice that, even in a vacuum for which *μ*=1, *ϵ*=1, the speed of propagation of the waves is finite, *c*=(*ϵ*_0_*μ*_0_)^−1/2^. Maxwell used Weber and Kohlrausch's experimental values for the product *ϵ*_0_*μ*_0_ and found, to his amazement, that *c* was almost exactly the speed of light. In Maxwell's letters of 1861 to Michael Faraday and William Thomson, he showed that the values agreed within about 1%. In Maxwell's own words, with his own emphasis, in the third part of his series of papers in *Philosophical Magazine*:

The velocity of transverse modulations in our hypothetical medium, calculated from the electro–magnetic experiments of MM. Kohlrausch and Weber, agrees so exactly with the velocity of light calculated from the optical experiments of M. Fizeau that we can scarcely avoid the inference that *light consists in the transverse modulations of the same medium which is the cause of electric and magnetic phenomena*. [20, p. 22]

This remarkable calculation represented the unification of light with electricity and magnetism.

Maxwell was fully aware of the remarkable mechanical basis for his model of the vacuum which had provided the inspiration for his discovery. As he wrote [19, p. 346],

The conception of a particle having its motion connected with that of a vortex by perfect rolling contact may appear somewhat awkward. I do not bring it forward as a mode of connection existing in Nature … It is however a mode of connection which is mechanically conceivable and it serves to bring out the actual mechanical connections between known electromagnetic phenomena.

No one could deny Maxwell's virtuosity in determining the speed of light from his mechanical model of electromagnetic forces.

## 5. Act 4. Maxwell on ‘A dynamical theory of the electromagnetic field’ (1865)

Maxwell was well aware of the fact that the theory as propounded in his paper ‘On Physical lines of force’ was ‘awkward’ and considered it only a ‘provisional and temporary hypothesis’. The mechanical basis of his insights could be misunderstood, or even ridiculed, but they had set Maxwell off on the right track towards the definitive theory of the electromagnetic field. He therefore set about recasting the theory on a much more abstract basis without any special assumptions about the nature of the medium through which electromagnetic phenomena are propagated. In 1865, he published his great paper entitled ‘A dynamical theory of the electromagnetic field’ [1]. To quote Whittaker [25, vol. 1, p. 255]:

In this, the architecture of his system was displayed, stripped of the scaffolding by aid of which it had been first erected.

Interestingly, there is only one, somewhat apologetic, reference in a footnote to his papers ‘On physical lines of force’ of 1861–1862 in this paper.

As Siegel has pointed out [6,26, pp. 187–188], from the beginning of his researches into electromagnetism, he had three goals:

1. ‘first, to develop a theory that would encompass all the known facts of electricity and magnetism;
2. second, to accomplish this unification by using Michael Faraday's approach to electricity and magnetism – the *lines of force* or *field* approach;
3. and third, to have the new theory go beyond what was known from experiment …’

In this third goal, he envisaged predictions of the theory which could be confronted by future experiments, reflecting his deep interest in innovative experiment.

Maxwell's own view of the significance of this paper is revealed in what C.W.F. Everitt calls ‘a rare moment of unveiled exuberance’ in a letter to his cousin Charles Cay:

I have also a paper afloat, containing an electromagnetic theory of light, which, till I am convinced to the contrary, I hold to be great guns. [7, p. 203]

The paper of 1865 is lengthy and divided into seven parts. All of them contain insights and innovations which illustrate how his thinking had developed since his papers of 1861–1862. In this essay, I have translated Maxwell's notation into more familiar symbols for the sake of clarity of the argument for the contemporary reader—these translations are shown in table 1. Note that the set of equations is written in Maxwell's mixture of electrostatic and electromagnetic units, that is, in unrationalized units.^5^

**Table 1.** Translation table from Maxwell's notation to unrationalized units.

quantity							
mass of body	C			→	*M*_C_		
forces acting at A and B	A	B		→	*F*_A_	*F*_B_	
velocities	*u*	*v*	*w*	→	*v*_A_	*v*_B_	*v*_C_
reduced masses ≡ inductances	L	M	N	→	*L*_A_	*M*_AB_	*L*_B_
resistances	R	S		→	*R*_A_	*R*_B_	
electromotive force acting on a circuit	*ξ*	*η*		→	*ε*_A_	*ε*_B_	
current flowing in a circuit	*x*	*y*		→	*I*_A_	*I*_B_	
quantity of electricity	X	Y		→	*Q*_A_	*Q*_B_	
heat generated by resistance	H			→	*H*		
intrinsic energy of currents	E			→	*E*		
work done when inductances change	W			→	*W*		
angular frequency	*p*			→	*ω*		
voltages	A	C	D	→	*V*_A_	*V*_C_	*V*_D_
electromagnetic momentum	F	G	H	→	*A*_*x*_	*A*_*y*_	*A*_*z*_
magnetic intensity	*α*	*β*	*γ*	→	*H*_*x*_	*H*_*y*_	*H*_*z*_
electromotive force	P	Q	R	→	*E*_*x*_	*E*_*y*_	*E*_*z*_
current due to true conduction	*p*	*q*	*r*	→	*J*_*x*_	*J*_*y*_	*J*_*z*_
electric displacement	*f*	*g*	*h*	→	*D*_*x*_	*D*_*y*_	*D*_*z*_
total current	*p*′	*q*′	*r*′	→	*J*′_*x*_	*J*′_*y*_	*J*′_*z*_
quantity of free electricity	*e*			→	*ρ*_e_		
electric potential	*Ψ*			→	*ϕ*		
magnetic potential	*ϕ*			→	*ϕ*_m_		
specific resistance	*ρ*			→	*ϱ*		
specific capacity of electric inductance	*D*			→	*ϵ*		

### (a) Part I—introductory

Maxwell begins with a lengthy introduction in which he reviews the entire field of electromagnetism and sets out with great clarity the agenda he proposes to follow in the paper. In fact, this introduction itself would find an appropriate place in any introductory undergraduate course on electromagnetism. At the outset, he emphasizes the difference in approach of his theory as compared with those of Weber and Neumann. Their approaches involved the use of ‘action at a distance’ without any account of how forces are transmitted from their source to other bodies. Maxwell states that he

… preferred to seek an explanation of the fact in another direction, by supposing them to be produced by actions which go on in the surrounding medium as well as in the excited bodies …

(3) The theory I propose may therefore be called a theory of the *Electromagnetic Field*, because it has to do with the space in the neighbourhood of the electric and magnetic bodies, and it may be called a *Dynamical* Theory, because it assumes that in that space there is matter in motion, by which the observed electromagnetic phenomena are produced.

Central to his theory was the *elasticity* of the medium through which electromagnetic phenomena are to be propagated, leading to the concept of the *displacement current* as a necessary part of the theoretical apparatus. Equally important is the formalization of the processes of electromagnetic induction that can produce currents—these can result in dissipative energy loss in resistances and chemical dissociation in electrolytes.

Maxwell had therefore to assume that there is an *aetherial medium*

pervading all bodies, and modified only in degree by their presence; that the parts of the medium are capable to being set in motion by electric currents and magnets; that this motion is communicated from one part of the medium to another by forces from the connexions of these parts.

Maxwell needed to assume that the aether was a physical component of the Universe, his logic being set out with remarkable clarity in his contribution to the ninth edition of the *Encyclopaedia Britannica* in 1878 [27].

Then he contrasts his approach with that of other investigators such as Helmholtz and Thomson, who deduced the phenomenon of induction from their mechanical actions. Maxwell proposes to follow the opposite agenda—to deduce the mechanical actions from the laws of induction. To achieve this he needs the *general equations of the electromagnetic field*, which consist of 20 equations with 20 variables. The paper is to set out the origins of these equations and among many applications is the key result that electromagnetic radiation propagates at the speed of light—the unification of light and electromagnetic radiation. Maxwell acknowledges the inspiration of Faraday's paper ‘Thoughts on ray vibrations’ [28]:^6^

The electromagnetic theory of light as proposed by (Faraday) is the same in substance as that which I have begun to develop in this paper, except that in 1846 there was no data to calculate the velocity of propagation. …

### (b) Part II—on electromagnetic induction

Next, Maxwell embarks on a lengthy discussion of electromagnetic induction. The mechanical origin of his thought informs his approach to the determination of the forms of the equations of self and mutual inductances between two inductors. Here he employs his technique of working by analogy to remarkable effect. As he writes:

Now, if the magnetic state of the field depends on motions of the medium, a certain force must be exerted in order to increase or diminish these motions, and when the motions are excited they continue, so that the effect of the connexion between the current and the electromagnetic field surrounding it is to endow the current with a kind of momentum, just as the connexion between the driving-point of a machine and a fly-wheel endows the driving point with an additional momentum, which may be called the momentum of the fly-wheel reduced to the driving point. The unbalanced force acting on the driving-point increases this momentum, and is measured by the rate of its increase.

The inspiration for this approach came from Lagrange's *Méchanique Analytique*. Maxwell had studied Poisson's *Traité de Méchanique* at Edinburgh Univeristy and was familiar with the French school of analytic dynamics, particularly praising Lagrange's insights. He presents what he calls the *reduced momentum* associated with the action of forces *F*_A_ and *F*_B_ acting on a body at driving points A and B. Adding in the frictional forces, for which the coefficients of resistance are *R*_A_ and *R*_B_, the total forces acting at A and B, *F*_A_′ and *F*_B_′, are
5.1
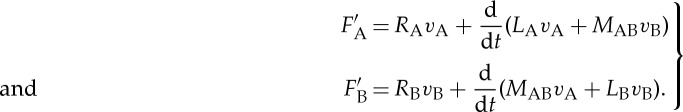

Note that Maxwell uses the identical symbols which will recur in the theory of self and mutual inductances and resistance for these mechanical effects. *F*_A_′ and *F*_B_′ are the forces referred to the points A and B, respectively. Then, in Maxwell's words,

If the velocity of A be increased at the rate d*v*_A_/d*t*, then in order to prevent B from moving a force, *F*′_B_=(d/d*t*)(*M*_AB_*v*_A_) must be applied to it.

This effect on B, due to an increase of the velocity of A, corresponds to the electromotive force on one circuit arising from an increase in the strength of a neighbouring circuit.

Of particular importance for Maxwell is this demonstration of *reduced momentum*, the quantities (*L*_A_*v*_A_+*M*_AB_*v*_B_) and (*M*_AB_*v*_A_+*L*_B_*v*_B_) in mechanics. The corresponding quantities in electromagnetic induction are the *electromagnetic momentum* or what Faraday referred to as the *electrotonic state*.

With this equivalence, Maxwell immediately converts the equations into the equations of induction between two current-carrying conductors. Then the equation for the current *I*_A_ in A is given by
5.2
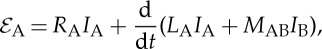

and that of *I*_B_ in B
5.3
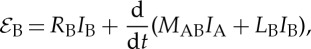

where 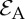 and 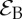 are the electromotive forces, *I*_A_ and *I*_B_ the currents, and *R*_A_ and *R*_B_ the resistances in A and B, respectively.

With these identifications, Maxwell proceeds to set out formally the processes of induction for one and two currents. From these, he derived the expressions for work and energy, the heat produced by the currents, the intrinsic energy of the currents and the mechanical action between conductors, as well as solving particular examples for one, two and six conductors. This last example was of practical importance since the six conductors could form an electric current balance for the practical measurement of inductances. Here as elsewhere in the paper, Maxwell provides the theory for the precise measurement of electrical quantities, which was a major concern to him, for example, to determine how accurately his theoretical value for the speed of light corresponded with the ratio of electrostatic to electromagnetic units of charge.

This part concludes with discussions of the *Exploration of the Electromagnetic Field*, *Lines of Magnetic Force* and *Magnetic Equipotential Surfaces*, the latter two topics being derived from his close reading of Faraday's *Experimental Researches in Electricity*. In the first, he describes experiments which illustrate how induced electromagnetic effects can be demonstrated and measured.

### (c) Part III—general equations of the electromagnetic field

In this part, the heart of the paper, the complete set of Maxwell's equations for the electromagnetic field are enunciated with extraordinary clarity and compelling logic. The equations as presented here are described by the alphabetical numbering used in his paper. In summary, these are:

*Three equations of magnetic force* (*H*_*x*_,*H*_*y*_,*H*_*z*_)

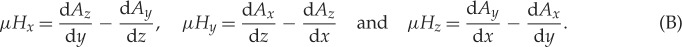



*Three equations of electric currents* (*J*_*x*_,*J*_*y*_,*J*_*z*_)

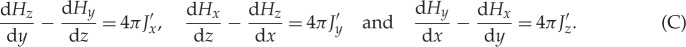



*Three equations of electromotive force* (*E*_*x*_,*E*_*y*_,*E*_*z*_)

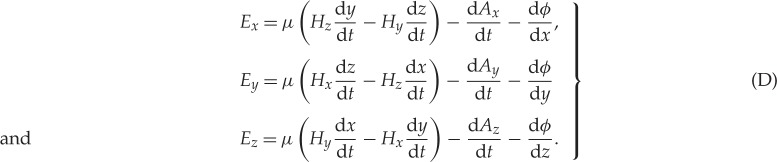



*Three equations of electric elasticity* (*D*_*x*_,*D*_*y*_,*D*_*z*_)

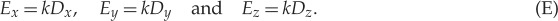



*Three equations of electric resistance* (*ϱ*)

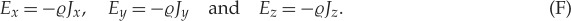



*Three equations of total currents* (*J*′_*x*_,*J*′_*y*_,*J*′_*z*_)

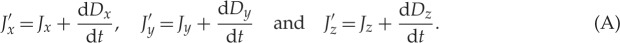



*One equation of free electricity* (*ρ*_*e*_)

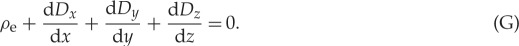



*One equation of continuity* (d*ρ*_*e*_/d*t*)

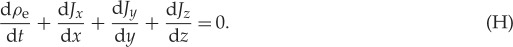

The result is 20 equations for the 20 variables which are:

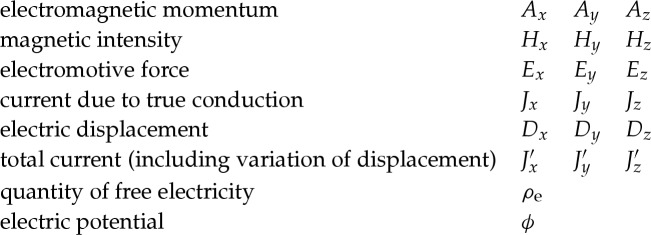

Written in contemporary notion, the equations have a familiar appearance. If we identify *μ****H*** with ***B***, the equations (A)–(H) can be written using vector operator notation as follows:

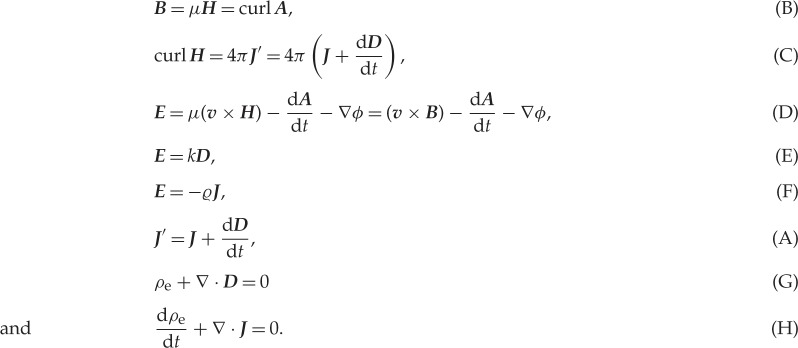

Thus,

Maxwell's first equation is found by taking the curl of (D) and omitting the ***v***×***B*** term.

Maxwell's second equation is (C).

Maxwell's third equation is (G).

Maxwell's fourth equation is found from the divergence of (B).

(A) is absorbed into (C).

(D) contains the expression for the (***v***×***B***) force on a particle, normally referred to as the Lorentz force.

(E) is the constitutive expression relating ***E*** and ***D***.

(F) is Ohm's law, which Maxwell recognized was only an empirical relation.

(H) is the continuity equation for electric charge.

Although Heaviside and Hertz are usually given credit for putting Maxwell's equations into their conventional form, it is apparent that only the simplest modifications were needed from Maxwell's original version. This would include a consistent use of Gaussian units, to be replaced by the SI system in 1960.

There are some intriguing features of the way in which Maxwell presents these equations.

— Whereas the electrical displacement (*D*_*x*_,*D*_*y*_,*D*_*z*_) appeared awkwardly in his papers of 1861–1862, it is now deeply embedded in the structure of electromagnetism in the second section of this part of the paper as
  the opposite electrification of the sides of a molecule or particle of a body which may or may not be accompanied with transmission through the body.
  The idle-wheels were an unnecessary artefact—the phenomenon of electrical displacement must necessarily occur and its time variation contributes to the total current.
— The electromagnetic momentum *A*_*x*_,*A*_*y*_,*A*_*z*_ is identified with what we would now call the vector potential. The origin of this identification is apparent from equation (3.8) above, but it also presages the four-vector notation of special relativity in which the four-vector for the four-potential is written [*ϕ*/*c*,*A*_*x*_,*A*_*y*_,*A*_*z*_]. Maxwell makes liberal use of the vector potential in his development of the equations, in contrast to contemporary practice of working initially with the fields ***E***,***D***,***B***,***H*** and ***J***.
— It is intriguing that Maxwell includes Ohm's law in his exposition of the structure of the equations, which is avoided in the contemporary set of equations. Maxwell was well aware of the empirical basis of Ohm's law in contrast to the equations of electromagnetic induction. As already mentioned, one of his first successful projects as Cavendish Professor was to establish Ohm's law with much improved precision.

The final section of this part of the paper is the determination of the various forms of energy associated with the fields. He starts again with the electromagnetic momentum, or vector potential, *A*_*x*_,*A*_*y*_,*A*_*z*_ and finds the total energy from the expression
5.4
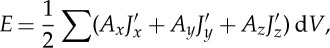

summing over all the space occupied by the currents. The total energy can be related to the total energy existing in the fields themselves:
5.5
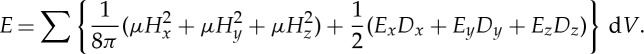



Maxwell then describes the key significance of this expression

(74) In speaking of the Energy of the field, however, I wish to be understood literally. All energy is the same as mechanical energy, whether it exists in the form of motion or in that of elasticity, or in any other form. The energy in electromagnetic phenomena is mechanical energy. The only question is, Where does it reside? On the old theories it resides in the electrified bodies, conducting circuits, and magnets, in the form of an unknown quality called potential energy, or the power of producing certain effects at a distance. On our theory it resides in the electromagnetic field, in the space surrounding the electrified and magnetic bodies, as well as in those bodies themselves, and is in two different forms, which may be described without hypothesis as magnetic polarization and electric polarization, or, according to a very probable hypothesis, as the motion and the strain of one and the same medium.

### (d) Part IV—mechanical actions in the field

Maxwell's next task is to show how these expressions for the various fields lead to the known laws of forces between the various electromagnetic entities. Note that these force laws had not been explicitly included in the formulation of the equations of electromagnetism. In this part, he derives from the field equations the known laws of force of a conductor moving through a magnetic field, the mechanical force on a magnet and the force on an electrified body.^7^

Maxwell also points out that the coefficient of ‘electric elasticity’ *k*, which appears in equation (E) of his equations is directly related to the ratio of electrostatic to electromagnetic units of measurement *v* through the expression *k*=4*πv*^2^. Weber and Kohlrauch had already determined this ratio experimentally and found it to be *v*=310 740 000 m s^−1^.

The last section of this part involves trying to apply the same techniques to understand the laws of gravity. Here, Maxwell hits the problem of the universal attractive force of gravity which results in negative values for the energy of a gravitating system. He confesses, ‘As I am unable to understand in what way a medium can possess such properties, I cannot go any further in this direction in searching for the cause of gravitation’.

### (e) Part V—theory of condensers

This section is concerned with the determination of the capacity and absorption of capacitors of various construction. This was an issue of considerable importance for the laying of long lengths of submarine telegraph cables, highlighted by the failure of the project to lay the first transatlantic telegraph cable in 1858.^8^ Maxwell had already been deeply involved in the issues concerning the determination of fundamental constants and of absolute standards of resistance. The 1861 meeting of the British Association had appointed a committee to oversee the determination of fundamental standards and Maxwell joined the Committee in 1862, soon after the publication of his papers on electromagnetism of 1861–1862.

He was deeply involved in testing his theory by precise experiment, in particular, the determination of the ratio of electrostatic to electromagnetic units of electric charge. The activities of the Committee became much more mathematical and theoretical, playing directly to Maxwell's strengths. He set about supervising the design and construction of apparatus to make a very accurate determination of the ohm with his colleagues Balfour Stewart and Fleeming Jenkin at King's College London [31]. The success of these experiments convinced the Committee that the absolute value of the ohm determined by this and similar techniques was the clearly preferred standard.

The work on standards fragmented in subsequent years, but Maxwell maintained his strong interest and leading role in the subject and made it one of the central themes of the research programme of the new Cavendish Laboratory in 1874. The result was that the work on determining the absolute standard of resistance was transferred from the Kew Observatory to the Cavendish. This work was to remain one of the central roles of the Cavendish until it was taken over by the National Physical Laboratory on its foundation in 1900.

### (f) Part VI—electromagnetic theory of light

Part VI is another memorable episode in this paper. Maxwell now seeks to determine whether or not the waves which can be propagated through any material medium are consistent with the postulate that light can be identified with electromagnetic waves. The analysis looks almost identical to that which appears in all modern standard texts on electromagnetism. Setting the conduction terms to zero, he derives the equations for the propagation of electromagnetic waves in the *x*,*y*,*z* directions in a matter of a page or so:
5.6
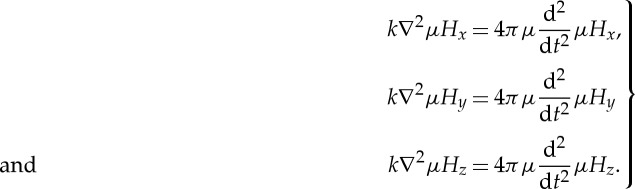



He continues

(95) If we assume that *H*_*x*_,*H*_*y*_,*H*_*z*_ are functions of *lx*+*my*+*nz*−*V* *t*=*w*, the first equation becomes
5.7
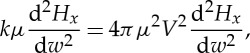

or
5.8
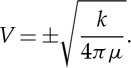

The other equations give the same value of *V*, so that the wave is propagated in either direction with a velocity *V*.

This wave consists entirely of magnetic disturbances, the direction of magnetization being in the plane of the wave. No magnetic disturbance whose direction of magnetization is not in the plane of the wave can be propagated as a plane wave at all.

Hence magnetic disturbances propagated through the electromagnetic field agree with light in this, that the disturbance at any point is transverse to the direction of propagation, and such waves may have all the properties of polarized light.

For the case of air, for which *μ*=1, the speed of propagation of light was measured by Foucault to be 298 000 000 m s^−1^ compared with the value derived by Weber and Kohlrausch who found *v*=310 740 000 m s^−1^ from their measured value of *k*. These figures also agreed within experimental error with the value of the speed of light determined from the astronomical aberration of light rays.

Next, Maxwell goes on to show that the refractive index of a non-conducting material *n* is given by the square root of the specific inductive capacity of the medium. He writes

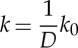
 and 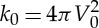 … Hence
5.9
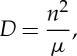

or the Specific Inductive Capacity is equal to the square of the index of refraction divided by the coefficient of magnetic induction.

Maxwell then makes a preliminary exploration of the case of anisotropic crystalline materials. He was well aware of the fact that crystals could have different refractive indices along different axes and so studies the case in which *μ* takes different values along the *x*,*y*,*z* directions.

The next application is the relation between electrical resistance and the transparency of materials. The objective was to account for the fact that, as he put it, ‘Most transparent solid bodies are good insulators, whereas all good conductors are very opaque.’ He investigates the propagation of light along the *x*-axis of the transverse disturbance *A*_*y*_. Including the resistivity *ϱ* of the medium, the propagation equation becomes
5.10
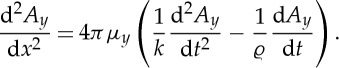

If *A*_*y*_ takes the form
5.11
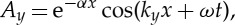

where *α* is the absorption coefficient,
5.12
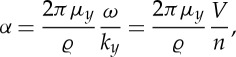

where *V* is the velocity of light in air, and *n* is the index of refraction. The proportion of incident intensity of light transmitted through the thickness *x* is
5.13
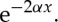

If *R* is the resistance of a sample of the material of thickness is *x*, breadth *b* and length *l*, then
5.14
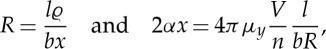

establishing quantitatively the relation between *α*, *n* and *R*.

### (g) Part VII—calculation of the coefficients of electromagnetic induction

The final part of the paper concerns the accurate estimation of the coefficients of electromagnetic induction. This might seem a descent from the heights of parts III, IV and VI of the paper, but these calculations were of central importance for the absolute determination of the ohm, one of Maxwell's preoccupations for the rest of his life. Suffice to say that Maxwell describes in some detail the various ways in which self and mutual inductances could be measured, in the context of the experiments which he and his colleagues were carrying out at King's College London and which were contained in the Report to the Committee of the British Association. The considerations were to find their application in the meticulous experiments of Rayleigh and colleages [32,33] in their determination of the absolute value of the ohm after Maxwell's death.

## 6. Finale—the aftermath

The identification of light with electromagnetic radiation was a triumph, providing a physical foundation for the wave theory of light, which could successfully account for the phenomena of reflection, refraction, polarization and so on. It is striking, however, how long it took for Maxwell's deep insights to become generally accepted by the community of physicists. He elaborated the theory in his great *Treatise on Electricity and Magnetism* as soon as he had settled back at his home at Glenlair in the Dumfries and Galloway region of southern Scotland in 1865.

It is significant that, while he was writing the *Treatise*, he was also an examiner for the Cambridge Mathematical Tripos and realized the dire need for suitable textbooks. The two-volume *Treatise* is, however, unlike many of the other great treatises such as Newton's *Principia* in that it is not a systematic presentation of the subject but a work in progress, reflecting Maxwell's own approach to these researches. In a later conversation, Maxwell remarked that the aim of the *Treatise* was not to expound his theory finally to the world, but to educate himself by presenting a view of the stage he had reached. Disconcertingly, Maxwell's advice was to read the four parts of the *Treatise* in parallel rather than in sequence.

The advantage of this approach was that Maxwell laid out clearly his own perception of the physical content of the theory and also how it could be confronted by precise experiment. These topics were strongly influenced by his work for the British Association committee on fundamental standards. He devotes a large part of the *Treatise* to basic measurements and electrical apparatus, in the process providing many experimental challenges which would be taken up by the research students in the Cavendish Laboratory. For example, he analyses five methods of making absolute determinations of the standard of resistance, occupying the whole of ch. XVIII.

Then, on reaching section 585, halfway through Volume 2, Maxwell states that he is ‘to begin again from a new foundation without any assumption except those of the dynamical theory’ [16, vol. 2, p. 229]. As summarized by Peter Harman, Maxwell emphasizes the expression of physical quantities free from direct representation by a mechanical model. This needed new mathematical approaches to electromagnetism, including quaternions, integral theorems such as Stokes' theorem, topological concepts and Lagrangian–Hamiltonian methods of analytic dynamics.

One of the most important results appears in section 792 of Volume 2 in which Maxwell works out the pressure which radiation exerts on a conductor on the basis of electromagnetic theory. This profound result provides a relation between the pressure *p* and the energy density *ε* of radiation derived purely from the properties of electromagnetic fields, *p*=*ε*/3*c*^2^. This result was to be used by Boltzmann in his paper of 1884 in which he derived the Stefan–Boltzmann law from classical thermodynamics. Published in 1873, the *Treatise* had an immediate impact and, together with Thomson and Tait's *Treatise on Natural Philosophy*, provided students with a comprehensive overview of both experimental and theoretical physics.

Maxwell was remarkably modest about his contribution. As Freeman Dyson has noted, when Maxwell was President of the Mathematical and Physical Sciences Section of the British Association for the Advancement of Science in 1870, his presidential lecture was a splendid opportunity for describing his new ideas, and yet he scarcely mentioned his recent work on electromagnetism, simply referring in an off-hand way as, ‘Another theory of electricity which I prefer’, without even mentioning that it was his own. As Dyson [3, p. 3 remarks], ‘The moral of this story is that modesty is not always a virtue’.

But the problems were much deeper. Not only was Maxwell's theory complex, but the discovery of the equations for the electromagnetic field also required a major shift in perspective for physicists of the late nineteenth century. It is worth quoting Dyson a little further.

There were other reasons, besides Maxwell's modesty, why his theory was hard to understand. He replaced the Newtonian universe of tangible objects interacting with one another at a distance by a universe of fields extending through space and only interacting locally with tangible objects. The notion of a field was hard to grasp because fields are intangible. The scientists of that time, including Maxwell himself, tried to picture fields as mechanical structures composed of a multitude of little wheels and vortices extending throughout space. These structures were supposed to carry the mechanical stresses that electric and magnetic fields transmitted between electric charges and currents. To make the fields satisfy Maxwell's equations, the system of wheels and vortices had to be extremely complicated. If you try to visualize the Maxwell theory with such mechanical models, it looks like a throwback to Ptolemaic astronomy with planets riding on cycles and epicycles in the sky. It does not look like the elegant astronomy of Newton. [3, p. 3]

Maxwell died in 1879 before direct experimental evidence was obtained for the existence of electromagnetic waves. The matter was finally laid to rest ten years after Maxwell's death in a classic series of experiments by Heinrich Hertz, almost 30 years after Maxwell had identified light with electromagnetic radiation. Hertz's great monograph *On Electric Waves* [34] sets out beautifully his remarkable set of experiments.

**Figure 3.**
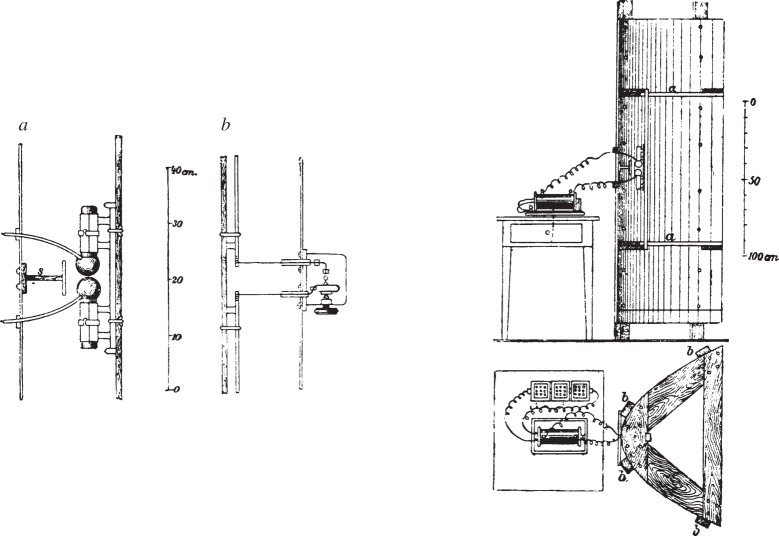
Hertz's apparatus for the generation and detection of electromagnetic radiation. The emitter *a* produced electromagnetic radiation in discharges between the spherical conductors. The detector *b* consisted of a similar device with the jaws of the detector placed as close together as possible to achieve maximum sensitivity. The emitter was placed at the focus of a cylindrical paraboloid reflector to produce a directed beam of radiation. (From [34].)

Hertz found that he could detect the effects of electromagnetic induction at considerable distances from his apparatus. Examples of the types of emitter and detector which he used are shown in figure 3. Electromagnetic radiation was emitted when sparks were produced between the large spheres by the application of a high voltage from an induction coil. The method of detection of the radiation field was the observation of sparks in the gap between the pair of small spheres of the detector.

After a great deal of trial and error, Hertz found that for particular arrangements there was a strong resonance, corresponding to the resonant frequencies of the emitter and detector. The frequency of the waves at resonance could be found from the resonant frequency of the emitter and detector which he took to be *ω*=(*LC*)^−1/2^, where *L* and *C* are the inductance and capacitance of the dipole. He measured the wavelength of the radiation by placing a reflecting sheet at some distance from the spark gap emitter so that standing waves were set up along the line between the emitter and the sheet. The speed of the waves could be found from the relation *c*=*ν*λ.

The speed turned out to be almost exactly the speed of light in free space. He then began a series of experiments which demonstrated conclusively that these waves behaved in all respects exactly like light—rectilinear propagation, polarization, reflection, refraction. Some of the experiments were quite remarkable in their execution. To demonstrate refraction he constructed a prism weighing 12 cwt out of so-called ‘hard pitch, a material like asphalt’.

The experiments demonstrated convincingly that there exist electromagnetic waves of frequency approximately 1 GHz and wavelength 30 cm which behaved in all respects like light. These great experiments were conclusive proof of the validity of Maxwell's equations.

## Footnotes

1 Note that the date on the scanned version of the paper on the Royal Society's website is incorrect (1 January 1865). This error is an artefact of the way the article had been digitized.

2 For further reading about Maxwell's life and science, see [3,5–12].

3 According to Dr David Ritchie of the James Clerk Maxwell Foundation, ‘[Maxwell] adopted the term ‘curl’ to describe two dimensional electrical and magnetic waves. He said they are not a ‘twirl’ which would imply three dimensions.’ There is a delightful watercolour by Jemima Wedderburn showing children playing on the frozen loch at St Mary's Isle on 23 December 1853 (figure 1). In the background, the adults can be seen curling, Maxwell presumably being among them, at home for the Christmas break from Cambridge. The rotation of the curling stones is two dimensional.

4 I give a number of examples of these in my book *Theoretical Concepts in Physics*[24, ch. 5].

5 More details of the various systems of units used in electromagnetism can be found in the essay *Unit Systems in Electromagnetism* at http://info.ee.surrey.ac.uk/Workshop/advice/coils/unit_systems/.

6 This paper was first published in *Philosophical Magazine*[29], following his lecture at the Royal Institution in April 1846.

7 Many parts of this analysis are similar to those which I described in my book [24, ch. 6]. There I started with Maxwell's equations as a set of partial differential equations and then gave them physical significance by carrying out various vector manipulations to show that all the known phenomena of electromagnetism could be derived from the postulated set of equations. Until I wrote the present essay, I had not appreciated that this approach, which we taught in a theoretical physics examples class in Cambridge, was almost exactly what Maxwell had published in 1865.

8 The history of the laying of the transatlantic cable and its importance for promoting the use of theoretical physics techniques as applied to electrical engineering projects is splendidly told by C. Smith and N. M. Wise in their biography of William Thomson [30].

## Author profile

**Malcolm Longair** is Jacksonian Professor Emeritus of Natural Philosophy, University of Cambridge and Director of Development at the Cavendish Laboratory, Cambridge. From 1980 to 1990, he was Astronomer Royal for Scotland and Director of the Royal Observatory, Edinburgh. From 1997 to 2005, he was Head of the Cavendish Laboratory. His research interests are in high energy astrophysics and astrophysical cosmology. Recent activities have included the scientific history of physics, astronomy, astrophysics and cosmology. In 2013, he published a history of quantum mechanics and is currently writing a scientific history of the Cavendish Laboratory.

## Appendix A

In a linear elastic medium, the displacement of the electric particles is proportional to the electric field strength

A 1

When the strength of the field varies, the charges move, causing what Maxwell called a *displacement current*. If *N*_q_ is the number density of electric particles and *q* the charge on each of them, the *displacement current density* is

A 2

Maxwell argued that this displacement current density should be included in the equation *curl* ***H***=***J*** which should now read

A 3

At this stage *α* and *β* are unknown constants to be determined from the known electric and magnetic properties of the medium.

First of all, let us work out the speed of propagation of a disturbance through the medium. Assuming that there are no currents, ***J***=0, the first two Maxwell's equations reduce to

A 4

The *dispersion relation* for these waves, that is, the relation between the wavevector ***k*** and the angular frequency *ω*, can be found by the standard procedure. We seek wave solutions of the form e^i(***k***⋅***r***−*ωt*)^, and so replace the vector operators by scalar and vector products according to the prescription:

Then equations (A 4) reduce to

A 5

Eliminating ***E*** from equations (A 5),

A 6

where we have used the linear constitutive relation ***B***=*μμ*_0_***H***, *μ* being the permeability of the medium. Using the vector relation ***A***×(***B***×***C***)=***B***(***A***⋅***C***)−***C***(***A***⋅***B***), we find that

A 7

There is no solution for ***k*** parallel to ***H***, that is, *longitudinal waves*, since the left-hand side of (A 6) is then zero. There exist solutions, however, for *transverse waves*, for which ***k***⋅***H***=0. These represent plane transverse waves with the ***E*** and ***H*** vectors perpendicular to each other and to the direction of propagation of the wave. The dispersion relation for the waves is thus *k*^2^=*ω*^2^*βμμ*_0_. Since the speed of propagation of the wave is *c*=*ω*/*k*, we find that

A 8

Notice that, because of the linear proportionality of *k* and *ω*, the phase velocity, *c*_p_, and the group velocity of a wave packet, *c*_g_=*dω*/d*k*, both have the same value, *c*, given by (A 8).

Now, Maxwell knew how to evaluate the constant *β*. The energy density stored in the dielectric is just the work done per unit volume in displacing the electric particles a distance ***r***, that is,

A 9

But

A 10

and hence

A 11

Therefore, the work done is

A 12

But this is equal to the electrostatic energy density in the dielectric, which is , where *ϵ* is the permittivity of the medium. Therefore, *β*=*ϵϵ*_0_. Inserting this value into (A 8) for the speed of the waves, we find

A 13
